# Radiation Dose Estimation and Lifetime Attributable Risk of Radiation-Induced Cancer from Chest CT in COVID-19 Patients

**DOI:** 10.1007/s11845-025-04046-8

**Published:** 2025-08-08

**Authors:** Husam H. Mansour, Noor Khairiah A. Karim, Noor Diyana Osman, Rohayu Hami, Yasser S. Alajerami

**Affiliations:** 1 Department of Biomedical Imaging, Advanced Medical and Dental Institute, Universiti Sains Malaysia, Bertam, 13200 Kepala Batas, Pulau Pinang Malaysia; 2 Medical Imaging Department, Applied Medical Sciences Faculty, Al Azhar University-Gaza, P.O. Box 1277, Gaza City, Palestine; 3 Department of Community Health, Advanced Medical and Dental Institute, Universiti Sains Malaysia, Bertam, 13200 Kepala Batas, Pulau Pinang Malaysia

**Keywords:** Chest, COVID-19, Radiation-induced cancer, Spiral Computed Tomography

## Abstract

**Background:**

Computerized tomography (CT) scans for COVID-19 diagnosis have increased throughout the pandemic. This growth has raised concerns about the potential radiation-induced cancer risk. This study aimed to estimate the effective dose (ED) and lifetime attributable risk (LAR) of cancer incidence and mortality associated with a single non-contrast chest CT scan for COVID-19 pneumonia diagnosis.

**Methods:**

A retrospective analysis included 522 consecutive COVID-19 patients who underwent a single non-contrast chest CT at Gaza Strip hospitals (September 1, 2020–September 30, 2022). The ImPACT CT Dosimetry spreadsheet (Version 1.0.4) was used to estimate organ and effective doses. The XrayRisk.com calculator was used for calculating the LAR of cancer incidence from a single chest CT.

**Results:**

The study analyzed non-contrast chest CT scans of 522 patients, with a mean age of 50.9 ± 15.8 years, and 239 males. The mean ED was 3.6 mSv, with the highest organ doses in the lungs and breast. Female patients were at a higher risk of cancer, with a higher risk in younger age groups. The mean LAR of lung cancer incidence was 5.8 per 100,000 males, while breast cancer incidence was 6.6 per 100,000 females. The whole-body ED of a single non-contrast chest CT is equivalent to 12 chest radiography series and three mammography screenings, or approximately four months of natural background radiation.

**Conclusion:**

Chest CT scans for COVID-19 patients carry a low but measurable cancer risk, particularly for younger and female patients. The long-term health impacts of such exposure should be closely monitored.

## Introduction

In December 2019, Coronavirus Disease (COVID-19) was first identified following an outbreak of the novel and pathogenic severe acute respiratory syndrome coronavirus 2 (SARS-CoV-2) in Wuhan, Hubei Province, China [1]. Reverse transcription polymerase chain reaction (RT-PCR) was subsequently recommended as the gold standard for confirming COVID-19 infection [2]. However, the initial RT-PCR positivity rate for throat swab samples was estimated to be between 30 and 60%, likely due to sampling errors and limitations in test sensitivity [3]. Consequently, RT-PCR has limitations as a screening tool for identifying COVID-19 in infected populations.

As a result of the public health emergency, chest computed tomography (CT) imaging has been widely employed for initial diagnosis, monitoring lesion progression, and evaluating treatment response in COVID-19 patients [4]. Pneumonia, a common manifestation of COVID-19 that affects the respiratory system, is frequently observed on chest CT scans [5]. Previous studies have reported that some symptomatic patients tested negative for COVID-19 using RT-PCR but exhibited radiological signs of pneumonia on CT imaging [4, 6]. A meta-analysis reported a pooled sensitivity of 94%; however, the lower disease prevalence in countries outside China has raised concerns about the predictive value of chest CT in other settings [7].

The radiation risk associated with CT scans must be carefully considered, particularly in patients under 40 years of age. Multiple CT scans were frequently performed to monitor disease progression on average, each patient underwent 4.1 scans (range: 3 to 6) over a period of 4.1 days (range: 1 to 8 days) [8]. This practice raised concerns among healthcare professionals; eight practitioners expressed serious apprehension about the cumulative radiation exposure from repeated imaging, especially in younger individuals.

In the Gaza Strip, diagnostic imaging, particularly CT, is limited by constrained healthcare infrastructure. Despite these challenges, the availability of modern CT equipment enables optimization of radiation doses without compromising diagnostic accuracy [9]. This study aims to estimate the lifetime attributable risk (LAR) of cancer incidence and mortality associated with a single non-contrast chest CT scan used in the diagnosis of COVID-19 pneumonia. It also seeks to raise awareness among healthcare professionals and the general public regarding radiation exposure in COVID-19 patients and emphasize the importance of dose optimization.

## Method

### Study population

**Fig. 1:**
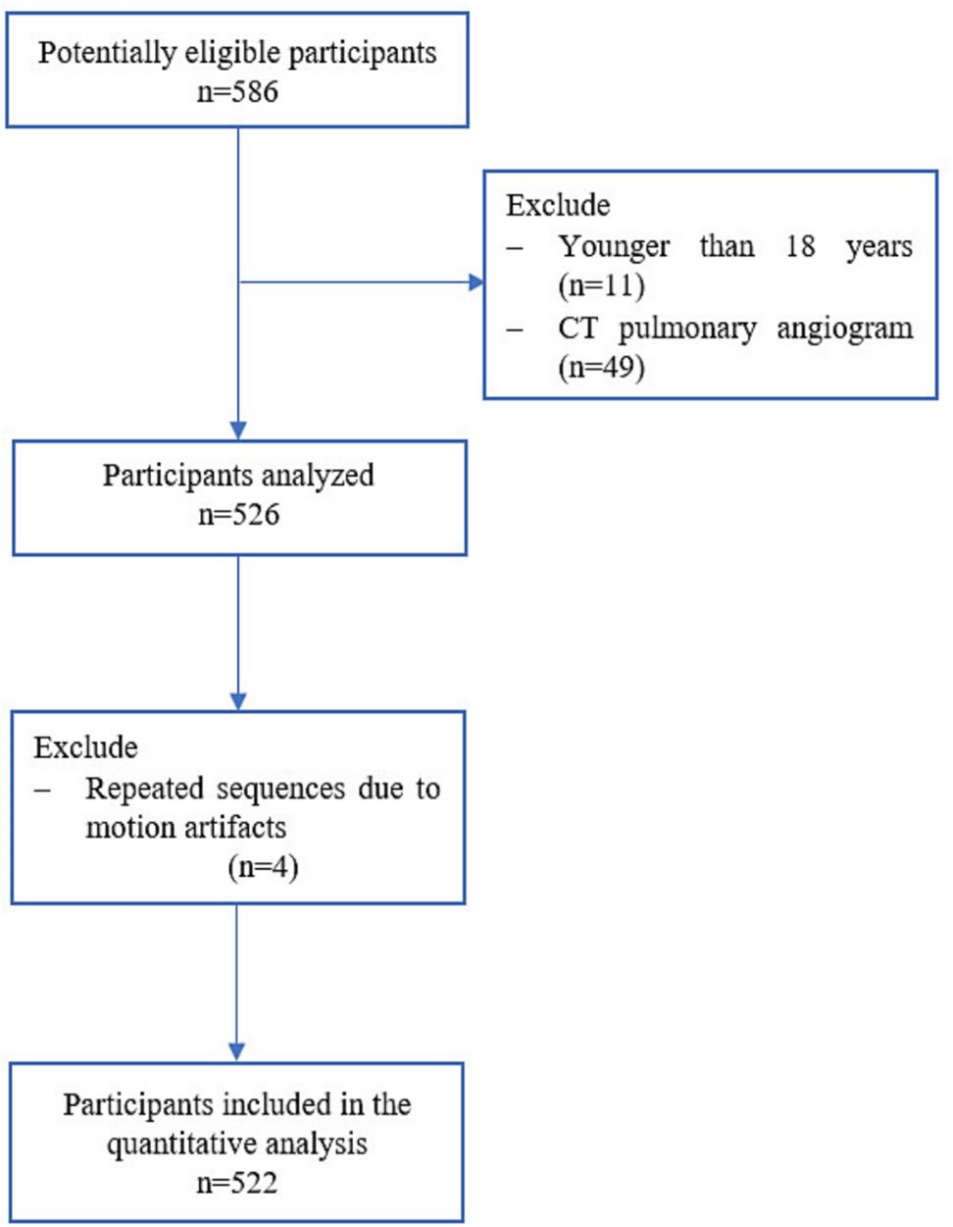
Flowchart illustrating the selection and inclusion process of the study population

This retrospective observational study on radiation dose included 522 consecutive patients with suspected or confirmed COVID-19 infection who underwent both RT-PCR testing and a non-contrast chest CT between September 1, 2020, and September 30, 2022, at Gaza Strip hospitals. The study included adult patients (≥ 18 years) who had a single chest CT scan. Data collection encompassed patient characteristics and technical parameters of the scans. Patients younger than 18 years, those who underwent CT pulmonary angiography, and cases with repeated sequences due to motion artifacts were excluded (Fig. 1).

### Chest CT acquisition

Chest CT scans were acquired using a Philips Brilliance 64 CT scanner. Technical parameters included a tube voltage of 120 kV and automatic tube current modulation based on patient body weight, ranging from 160 to 310 mA. The gantry rotation time was 0.5 s, with collimation of 64 × 0.625 mm (beam width of 40 mm), a pitch of 1, and a matrix size of 512 × 512. An iterative reconstruction technique was employed to enhance spatial resolution and reduce radiation dose. Non-contrast images were obtained from the lung apex to base during breath-hold in the inspiratory phase. The effective mAs, CT dose index (CTDI), and dose-length product (DLP) were recorded from the post-examination dose report for each scan. Images were reconstructed using a high-resolution lung algorithm with a slice thickness of 0.625 mm.

### Effective radiation dose estimation

**Fig. 2:**
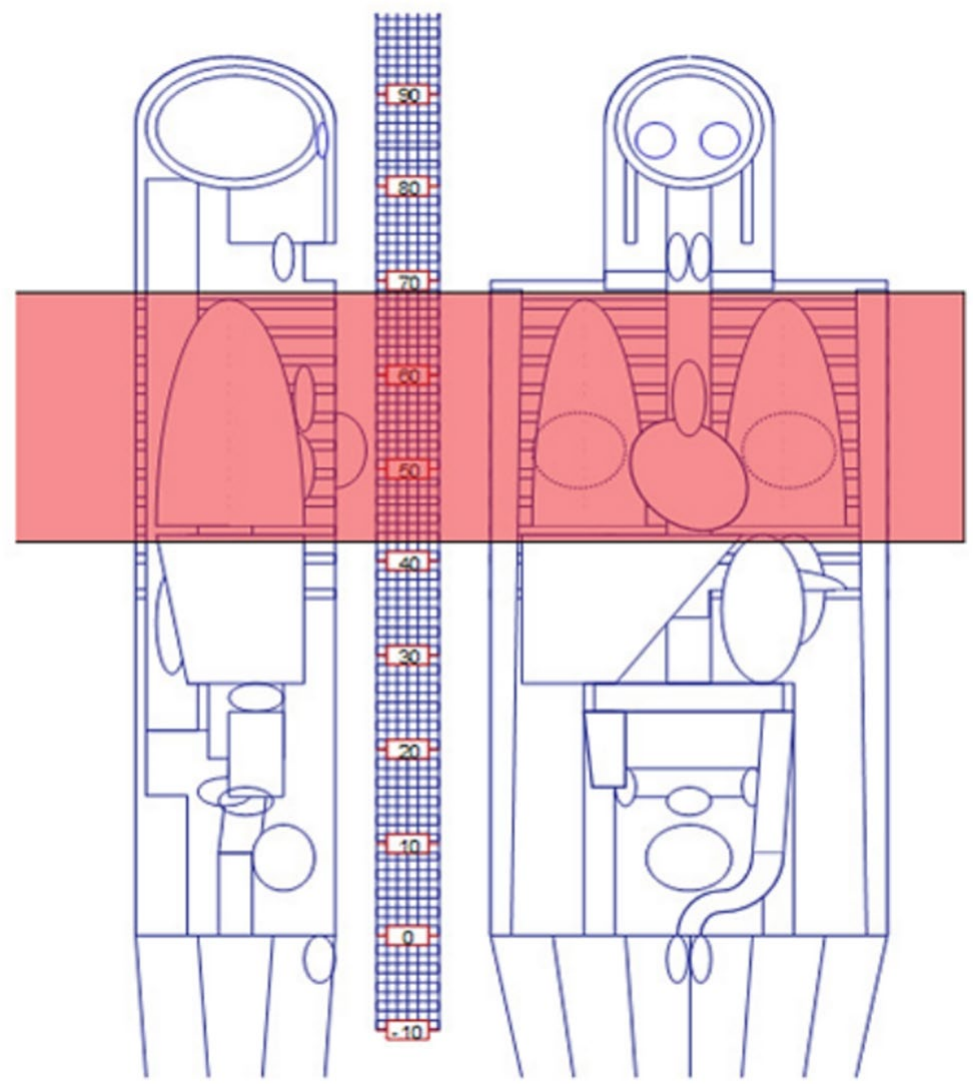
Adult hermaphrodite phantom model used in the ImPACT CT dosimetry software for organ dose estimation

The imPACT CT Dosimetry Spreadsheet (Version 1.0.4) was utilized to estimate organ and effective radiation doses from chest CT scans. This tool employs Monte Carlo dose datasets from the National Radiological Protection Board (NRPB), as detailed in Report SR250 by the Health Protection Agency’s Centre for Radiation, Chemical, and Environmental Hazards (Didcot, UK) [10]. SR250 provides normalized organ dose data for various CT scanners by simulating radiation exposure using a model Medical Internal Radiation Dose (MIRD) adult hermaphrodite phantom (Fig. 2). Organ doses were calculated using tissue weighting factors recommended by the International Commission on Radiological Protection (ICRP) Report 103 [11].

**Fig. 3:**
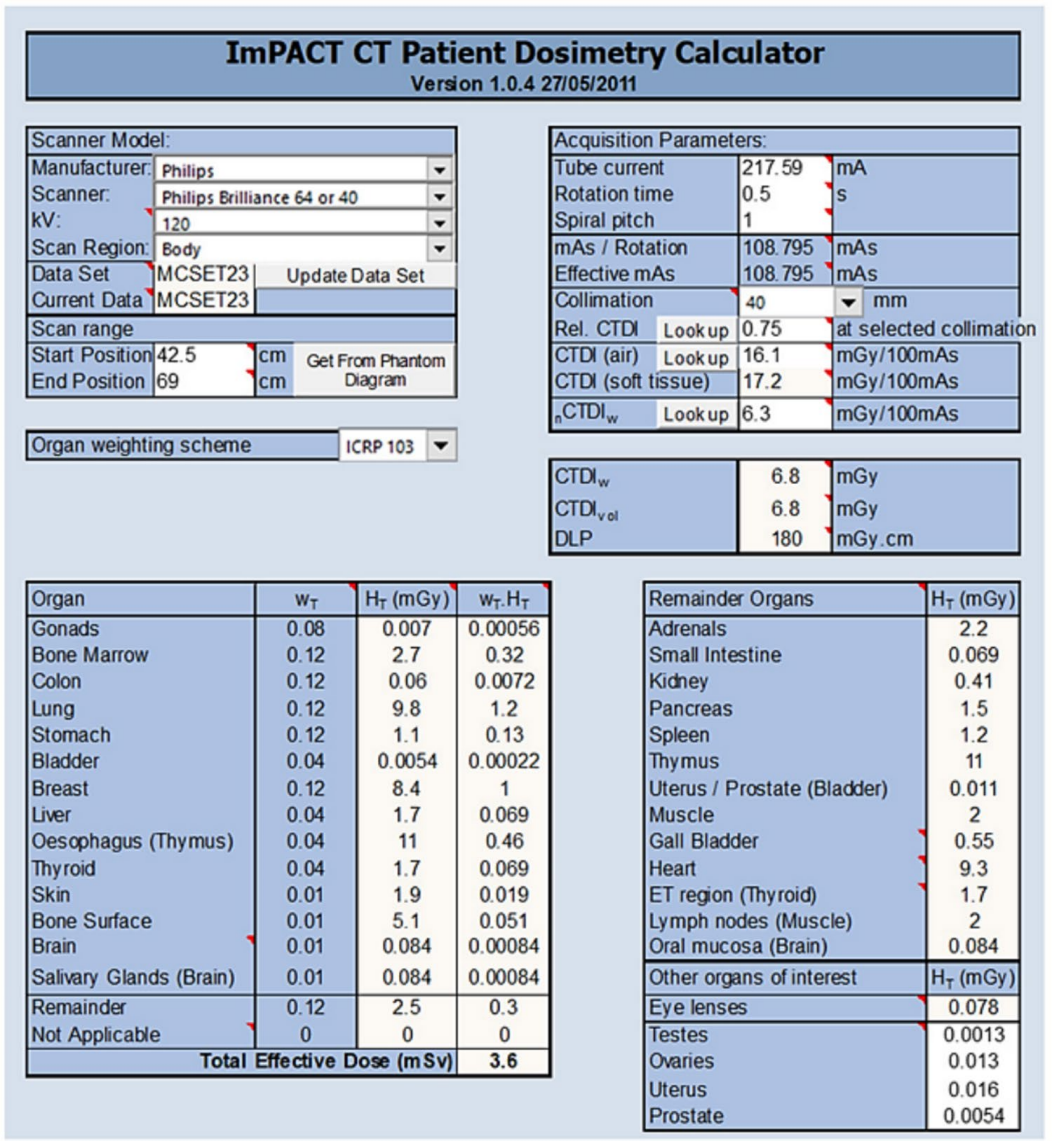
Overview of the ImPACT CT dosimetry spreadsheet used for estimating organ doses and effective radiation dose

The Monte Carlo dataset used to develop the ImPACT CT Dosimetry Spreadsheet includes CT dose index (CTDI) measurements in free air, at the center, and at the periphery of the phantom (CTDI100, P). A consistent ionization chamber and methodology were employed for all measurements, conducted within standard Perspex head and body dosimetry phantoms. These data are used to calculate the weighted CT dose index (CTDIw), volume CT dose index (CTDIvol), dose-length product (DLP), and other relevant dose parameters (Fig. 3). The dosimetry spreadsheet required manual entry of variable scan parameters, including gantry rotation time, tube current, and spiral pitch, which varied according to scanner technology and manufacturer. The equivalent organ dose (mGy) was estimated using the formula wT × HT, converting tissue weighting factors from ICRP Publication 103 into millisieverts (mSv) for compatibility with the ImPACT CT Dosimetry Spreadsheet.

### Lifetime attributable risk of cancer estimation

**Fig. 4:**
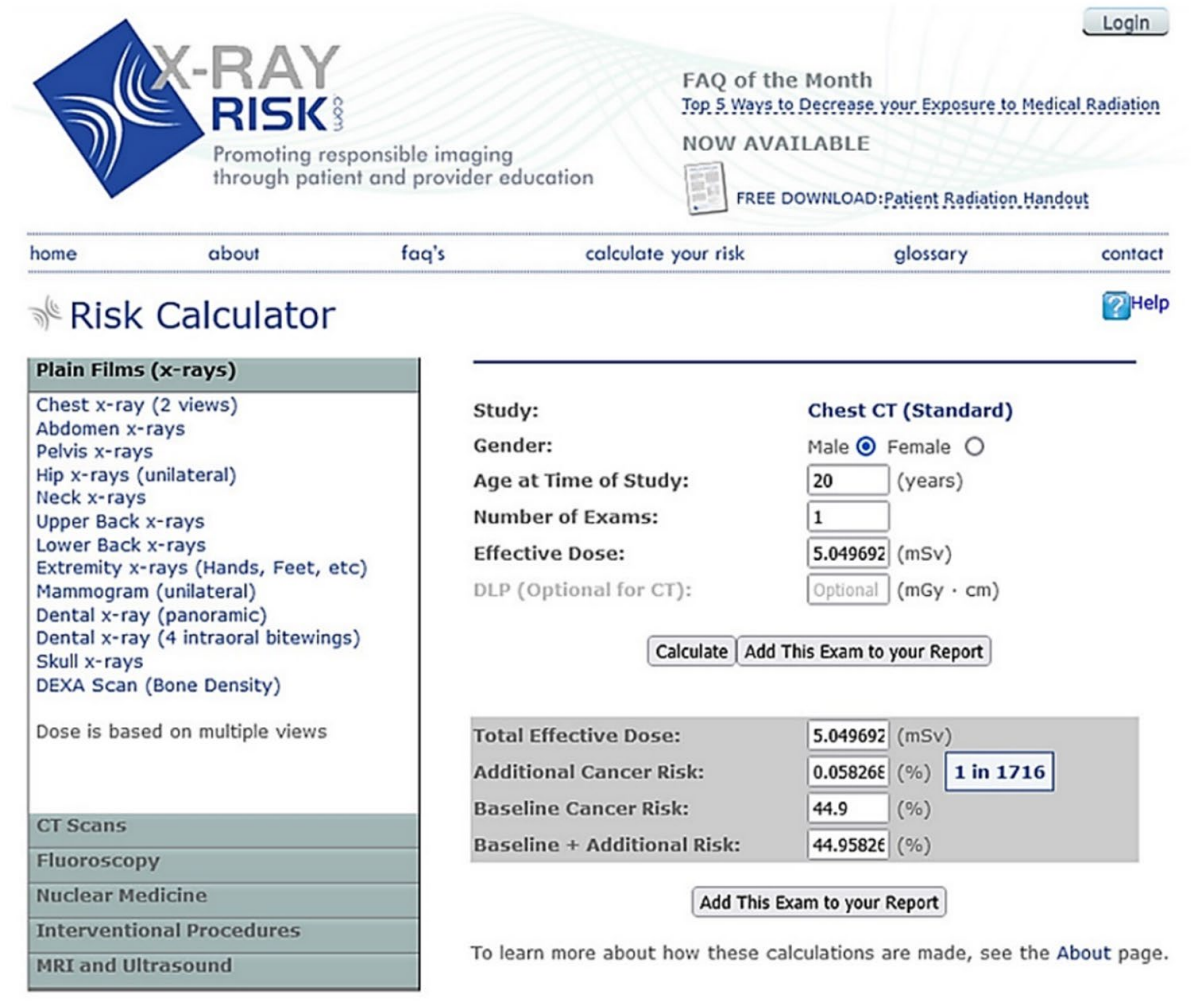
The XrayRisk.com online calculator used to estimate lifetime cancer risk from radiation exposure

The online calculator XrayRisk.com (Fig. 4), developed with support from the American Society of Radiologic Technologists (ASRT), was utilized to estimate the lifetime attributable risk (LAR) of cancer incidence and mortality associated with chest CT scans. XrayRisk.com was selected due to its user-friendly interface, validated predictive accuracy, and broad accessibility, making it appropriate for both clinical and research settings. This web-based tool calculates LAR based on variables including the scanned anatomical region, patient age, gender, and average radiation dose. It also incorporates cumulative radiation exposure over the patient's lifetime. LAR quantifies the additional risk of developing cancer above the background incidence. Notably, XrayRisk.com has been cited in several peer-reviewed studies for its utility and reliability in estimating radiation-related cancer risks in diagnostic imaging [12, 13].

### Statistical analysis

IBM SPSS Statistics software (version 23; IBM Corp., Armonk, NY, USA) was used for data analysis. Quantitative variables were summarized using the mean and standard deviation. The Pearson correlation coefficient (r) was applied to assess the relationship between patient age and the estimated lifetime attributable risk (LAR) of cancer incidence. A p-value < 0.05 was considered statistically significant.

## Results

### Patient characteristics

The characteristics of the patients and chest CT scan parameters are summarized in Table 1. Among the 522 patients, 239 (45.8%) were male and 283 (54.2%) were female. The age of patients ranged from 20 to 80 years, with a mean ± standard deviation (SD) of 50.9 ± 15.8 years, reflecting a broad demographic distribution. The mean volume CT dose index (CTDIvol) was 6.8 ± 1.2 mGy, and the mean dose-length product (DLP) was 180 ± 31 mGy·cm.

**Table 1:** Patient Characteristics and Chest CT Scan Parameters

Variable	Total (*n* = 522)
Age (y)^*^	50.9 ± 15.8 (20–80)
Gender	
Male	239 (45.8)
Female	283 (54.2)
Hospitalization period^*^	13.79 ± 8.15
Comorbidity	
Diabetes	173 (33.1)
Hypertension	131 (25.1)
Cardiomyopathy	93 (17.8)
Chest CT Scan Parameters	
Tube current (mA)	217.6 ± 37.9
Tube voltage^†^	120 kV
Gantry rotation time^†^	0.5 s
Pitch^†^	1
Collimation^†^	64 rows × 0.625 mm
CT dose index (CTDI)	6.8 ± 1.2
Dose-length product (mGy∙cm)	180 ± 31

Unless otherwise indicated, data are presented as mean ± standard deviation or as frequencies with percentages in parentheses

*Data are presented as mean ± standard deviation with ranges in parentheses

†Data represent number of participants

### Effective and organ dose estimations of patients'COVID chest CT

The total effective dose (ED) from chest CT scans ranged from 2.65 mSv to 5.13 mSv (mean ± SD = 3.6 ± 0.63 mSv). The lungs received the highest radiation dose (mean weighted equivalent dose: 1.172 ± 0.204 mSv), followed by the breast (mean: 1.004 ± 0.175 mSv). The next highest doses were recorded in the esophagus (0.459 ± 0.08 mSv) and bone marrow (0.321 ± 0.056 mSv) (Table 2).

**Table 2:** Effective and organ dose estimations of patients'COVID chest CT

Organ	Gender	Minimum Effective Dose	Maximum Effective Dose	Mean Effective Dose	Std. Deviation
Gonads	Male	0.00041	0.00079	0.00055	0.00010
	Female	0.00041	0.00080	0.00057	0.00010
	Overall	0.00041	0.00080	0.00056	0.00010
Bone marrow	Male	0.236	0.450	0.317	0.053
	Female	0.236	0.457	0.324	0.058
	Overall	0.236	0.457	0.321	0.056
Colon	Male	0.0053	0.0102	0.0072	0.0012
	Female	0.0053	0.0103	0.0073	0.0013
	Overall	0.0053	0.0103	0.0072	0.0013
Lung	Male	0.862	1.643	1.158	0.192
	Female	0.862	1.670	1.184	0.214
	Overall	0.862	1.670	1.172	0.204
Stomach	Male	0.094	0.180	0.127	0.021
	Female	0.094	0.183	0.130	0.023
	Overall	0.094	0.183	0.129	0.022
Bladder	Male	0.00016	0.00031	0.00022	0.00004
	Female	0.00016	0.00031	0.00022	0.00004
	Overall	0.00016	0.00031	0.00022	0.00004
Breast	Male	0.738	1.407	0.992	0.165
	Female	0.738	1.431	1.014	0.182
	Overall	0.738	1.431	1.004	0.175
Liver	Male	0.051	0.097	0.068	0.011
	Female	0.051	0.098	0.070	0.013
	Overall	0.051	0.098	0.069	0.012
Esophagus	Male	0.338	0.643	0.453	0.075
	Female	0.338	0.654	0.464	0.084
	Overall	0.338	0.654	0.459	0.080
Thyroid	Male	0.051	0.096	0.068	0.011
	Female	0.051	0.098	0.069	0.013
	Overall	0.051	0.098	0.069	0.012
Skin	Male	0.014	0.026	0.018	0.0030
	Female	0.014	0.026	0.019	0.0034
	Overall	0.014	0.026	0.019	0.0032
Bone surface	Male	0.038	0.072	0.051	0.0084
	Female	0.038	0.073	0.052	0.0093
	Overall	0.038	0.073	0.051	0.0089
Brain	Male	0.00062	0.00117	0.00083	0.00014
	Female	0.00062	0.00119	0.00085	0.00015
	Overall	0.00062	0.00119	0.00084	0.00015
Salivary gland	Male	0.00062	0.00117	0.00083	0.00014
	Female	0.00062	0.00119	0.00085	0.00015
	Overall	0.00062	0.00119	0.00084	0.00015
*Remainder Organs	Male	0.221	0.421	0.297	0.049
	Female	0.221	0.428	0.303	0.055
	Overall	0.221	0.428	0.30	0.052
Total Effective Dose (mSv)	Male	2.65	5.05	3.56	0.59
	Female	2.65	5.13	3.64	0.66
	Overall	2.65	5.13	3.60	0.63

*Remainder Organs: Adrenals, Small Intestine, Kidney, Pancreas, Spleen, Gall Bladder, Thymus, Muscle, Heart, Lymph nodes, Oral mucosa, Eye lenses, Uterus, Ovaries, Prostate, and Testes

### Cancer LAR for patients based on age group categories

A robust and statistically significant negative correlation was observed between patient age and the lifetime attributable risk (LAR) of cancer, as indicated by the Pearson correlation coefficient (*r* = −0.869, *p* < 0.001). On average, all patients had a 1 in 6002 chance of developing cancer. The cancer risk was higher among female patients, with an incidence of 1 in 5446, compared to male patients, whose incidence was 1 in 6660 following a chest CT. As shown in Table 3, the LAR for cancer was substantially higher in both males and females within the younger age groups.

**Table 3:** Cancer LAR comparison for male and female patients based on age group

Age group at exposure (years)	Gender	*N* = 522	LAR of cancer		
			Additional Cancer Risk (Mean)	Std. Deviation	(1 in chances)
20y to 30y	Male	22	0.000440	0.000111	2458 Male
	Female	33	0.000751	0.000119	1364 Female
31y to 40y	Male	49	0.000303	0.000034	3347 Male
	Female	56	0.000462	0.000066	2209 Female
41y to 50y	Male	56	0.000212	0.000024	4783 Male
	Female	48	0.000290	0.000034	3491 Female
51y to 60y	Male	43	0.000143	0.000014	7069 Male
	Female	54	0.000186	0.000023	5470 Female
61y to 70y	Male	42	0.000097	0.000011	10,436 Male
	Female	46	0.000129	0.000026	8011 Female
71y to 80y	Male	27	0.000075	0.000008	13,464 Male
	Female	46	0.000087	0.000015	11,761 Female
All Age groups	Male	239	0.000203	0.000115	6660 Male
	Female	283	0.000299	0.000216	5446 Female
	Overall	522	0.000255	0.000183	6002 Patients

## Discussion

While chest CT has played a vital role in diagnosing COVID-19 complications, the potential long-term radiation risks require careful consideration. The increased use of CT raises concerns about radiation-induced cancer, particularly among younger and more radiosensitive patients. Estimating radiation dose and lifetime attributable risk (LAR) underscores the necessity of dose optimization and prudent imaging justification, especially in resource-limited settings where repeated scans may be necessitated by diagnostic or follow-up challenges.

Various software tools have been developed to calculate organ doses in CT imaging. This study employed the ImPACT CT Dosimetry spreadsheet, which yielded a mean estimated effective dose of 3.6 ± 0.63 mSv. In a study by Čiva et al. [14], patients underwent scanning with a Toshiba Aquilion Lightning 16-row/32-slice CT scanner, and organ doses as well as effective doses were estimated using the Monte Carlo simulation-based CTVoxDos software. Their findings indicated a median effective dose of 2.6 mSv, which is lower than that reported in the present study. This discrepancy may be attributed to differences in the CT vendor, the number of detector rows, and the dose estimation software employed. Another investigation using Radimetrics software with pre-run Monte Carlo simulations reported an average effective dose of 4.4 mSv, exceeding that observed in the current study [15].

In 2008, the American Association of Physicists in Medicine reviewed multiple studies reporting an effective dose (ED) of approximately 0.014 mSv for chest CT. McCollough et al. (2008) calculated dose by multiplying the dose-length product (DLP) by the anatomical weighting factor, k [16]. Filatova et al. (2021) reported an average ED of 3.76 mSv for conventional CT [17]. Zhou et al. (2021) compiled radiation dose estimates from common chest CT protocols to establish diagnostic reference levels for COVID-19, with a median ED of 4.55 mSv [18]. Matkevich and Ivanov (2021) found average effective doses of 3.43 mSv in patients aged 19–64 and 3.28 mSv in those aged 65 and older [19]. These values align with a large-scale European assessment of adult CT diagnostic reference levels based on clinical indication [20].

The role of low-dose CT (LDCT) in diagnosing COVID-19 remains debated due to concerns that reduced image quality may affect diagnostic accuracy, particularly for subtle lung abnormalities. Although LDCT offers significantly lower radiation exposure, studies show variability in sensitivity and specificity compared to standard-dose CT. Homayounieh et al. (2021) reported that nearly half of centers across 28 countries lacked a standardized CT protocol for COVID-19 [21]. Kang et al. (2020) developed a low-dose protocol reducing median ED from 1.81 mSv to 0.203 mSv without noticeable loss of image quality [22]. Tabatabaei et al. (2020) found high inter-reader agreement (Kappa 0.80–0.84) between standard-dose (6.6 mSv) and LDCT (1.8 mSv), suggesting comparable image quality [23]. Dangis et al. [24] and Hamper et al. [25] confirmed LDCT’s accuracy in evaluating COVID-19 pneumonia, with effective doses under 1 mSv. Agostini et al. [26] demonstrated that LDCT and long-pitch chest CT using dual-source CT with spectral shaping (100 Sn kVp) provided high-quality diagnostic images with reduced radiation dose (0.28 mSv vs. 3.28 mSv) and fewer motion artifacts.

LDCT has not been widely accepted for COVID-19 follow-up, as noted by Shiri et al. [27], due to increased image noise. Reduced radiation dose limits the detection of ground-glass opacities characteristic of COVID-19 pneumonia. Their study found LDCT hindered recognition of lesions such as consolidation, crazy paving, nodular infiltrations, and broncho-vascular thickening in approximately 60% of cases. LDCT use is further limited by patient size variability and lack of advanced CT technologies, such as iterative reconstruction [21].

The International Atomic Energy Agency (IAEA) reports that 55% of COVID-19 CT imaging uses a standard chest CT protocol, 43% employ low-dose protocols, and 3% use high-dose protocols [28]. Cristofaro et al. [29] compared chest CT radiation doses between COVID-19 and lung infection patients from the previous year, finding higher mean doses in COVID-19 patients. Sakane et al. [30] indicated that a 5 mSv dose from standard chest CT may cause double-stranded DNA breaks and chromosomal abnormalities. However, the body can repair DNA damage and eliminate abnormal cells via apoptosis or other mitotic death processes [31].

While the current study estimated the effective dose (ED) for a single chest CT, literature reports indicate that an increasing number of patients are undergoing repeated chest scans, contributing to cumulative radiation exposure. In the study by Kang et al. [22], COVID-19 patients underwent two to five chest CT scans within a month in a clinical setting. Additionally, both hospitalized and non-hospitalized patients often require follow-up CT imaging to monitor pneumonia progression or resolution [32]. Selected individuals may even undergo multiple CT scans within a short timeframe to ensure clinical stability [33]. Therefore, greater attention should be paid to the medical radiation exposure associated with repeated imaging, particularly in vulnerable groups such as children and pregnant women, who are more sensitive to ionizing radiation.

In the context of lifetime attributable risk (LAR) of cancer incidence across all age groups, the current study found that the average additional cancer risk was approximately 1 in every 6,002 individuals (1 in 6,660 for males and 1 in 5,446 for females). This corresponds to a cancer risk of 25.5 per 100,000 people, with males exhibiting a risk of 20.3 per 100,000 and females 29.9 per 100,000. Notably, cancer risk decreased with increasing patient age. Among younger individuals (20–30 years), the estimated cancer risk was 44 per 100,000 for males and 75 per 100,000 for females. In contrast, the risk in the older age group (71–80 years) was 7.5 per 100,000 for males and 8.7 per 100,000 for females. The overall mean LAR for lung cancer incidence, based on organ-specific doses, was 5.8 per 100,000 in males (approximately 1 in 17,370), while the estimated breast cancer incidence in females was 6.6 per 100,000 (approximately 1 in 15,168).

Ghetti et al. [15] conducted an estimation of radiation doses and lifetime attributable risk (LAR) associated with chest CT during the COVID-19 pandemic, and their results are consistent with those of the current study. They reported that the LAR for all solid tumors was approximately 21 cases per 100,000 individuals. Notably, the likelihood of developing lung or breast cancer in women was 6.9 times higher than the risk of any other solid cancer. Similarly, in men, the risk of developing lung cancer was at least 2.3 times higher than that of any other cancer type.

A single chest CT scan performed in individuals with suspected COVID-19 may lead to an additional cancer case per 100,000 people [16]. The estimated lifetime attributable risk (LAR) varies by age group, with 24.1 cases per 100,000 in children, 23.3 in adolescents, 14.4 in adults, and 2.6 in the elderly. These differences may be attributed to variations in CT scanner specifications, scanning protocols, and radiation dose estimation methods. The referenced study used a Siemens SOMATOM Emotion 16 scanner with a pitch between 1.2 and 1.4. The dose-length product (DLP) was calculated exclusively for the chest region, from the lung apices to the bases. Moreover, their risk estimates were derived using a standardized conversion factor based on a 1 mSv exposure, which may further explain the observed discrepancies in radiation risk.

In this study, the effective dose (ED) of chest CT was compared with that of the two most commonly performed conventional radiology examinations. First, a standard posteroanterior and lateral chest radiograph series has an ED of approximately 0.1 mSv, equivalent to about 10 days of natural background radiation. Second, a screening mammography series, involving two views of each breast, has an ED of roughly 0.42 mSv, corresponding to around seven weeks of background radiation exposure [34]. These comparisons help contextualize CT radiation doses for both patients and clinicians.

Organ-specific dose analysis showed that a chest CT delivers a lung dose of approximately 1.172 mSv, equivalent to the dose from 12 chest radiograph series. The breast dose from a chest CT (1.014 mSv) is roughly equal to that from three mammography exams. One chest CT is also equivalent to about four months of background radiation exposure to the breast and lungs. Regarding whole-body exposure, a single chest CT (3.60 mSv) corresponds to the dose from 36 chest radiograph series or approximately one year of background radiation. In comparison to mammography, the ED from chest CT is equivalent to nine mammograms, or about 14 months of background radiation.

This study has several limitations concerning dose estimation and cancer risk assessment. One major limitation lies in the variability of patient size and tissue composition, which may affect the accuracy of dose estimation using the ImPACT software. This tool calculates organ doses based on a standard-sized phantom, which does not account for individual anatomical differences. Additionally, variations in CT protocols and scanner models across institutions and countries may further influence dose estimations and reduce generalizability. Nonetheless, the ImPACT software remains a widely accepted tool for estimating effective dose and has been extensively utilized in the literature for similar purposes [12, 35].

## Conclusion

In conclusion, this study underscores the need for heightened awareness regarding the potential cancer risks associated with chest CT imaging in the diagnosis and follow-up of COVID-19 pneumonia. By estimating the effective radiation dose (ED) and calculating the lifetime attributable risk (LAR) of cancer incidence and mortality, we demonstrate that although the radiation dose from a single chest CT scan is relatively low, repeated exposures, particularly in younger or more vulnerable populations, may pose a cumulative oncogenic risk. The findings also reveal significant variability in reported radiation doses across studies and highlight ongoing debates surrounding the implementation of low-dose CT protocols. These insights emphasize the importance of balancing diagnostic benefits with long-term radiation risks, advocating for judicious use of chest CT and the continued evaluation of its public health implications throughout and beyond the pandemic.

## Data Availability

The datasets used and/or analyzed during the current study are available from the corresponding author on reasonable request.
